# Delirium in geriatric patients

**DOI:** 10.1007/s10354-021-00904-z

**Published:** 2022-01-10

**Authors:** Bernhard Iglseder, Thomas Frühwald, Christian Jagsch

**Affiliations:** 1 Department of Geriatric Medicine, Salzburg University Hospital—Campus Christian-Doppler-Klinik, and Paracelsus Medical University, Ignaz-Harrer-Straße 79, 5020 Salzburg, Austria; 2 Specialist in Internal and Geriatric Medicine, Vienna, Austria; 3 Department of Geriatric Psychiatry and Geriatric Psychotherapy, State Hospital II, Graz, Austria

**Keywords:** Cognitive disorders, Acute confusional state, Prevention, Encephalopathy, Older people, Kognitive Störung, Akute Verwirrtheit, Prävention, Encephalopathie, Alte Menschen

## Abstract

Delirium is the most common acute disorder of cognitive function in older patients. Delirium is life threatening, often under-recognized, serious, and costly. The causes are multifactorial, with old age and neurocognitive disorders as the main risk factors. Etiologies are various and multifactorial, and often related to acute medical illness, adverse drug reactions, or medical complications. To date, diagnosis is clinically based, depending on the presence or absence of certain features. In view of the multifactorial etiology, multicomponent approaches seem most promising for facing patients’ needs. Pharmacological intervention, neither for prevention nor for treatment, has been proven effective unanimously. This article reviews the current clinical practice for delirium in geriatric patients, including etiology, pathophysiology, diagnosis, prognosis, treatment, prevention, and outcomes.

## Abstract (de)

Das Delir ist die häufigste akute Störung von kognitiven Funktion bei alten Patienten. Ein Delir ist lebensbedrohlich, wird häufig nicht erkannt und ist ebenso schwerwiegend wie kostspielig. Die Ursachen sind multifaktoriell, wobei das Alter und neurokognitive Erkrankungen die Hauptrisikofaktoren darstellen. Ätiologisch besteht häufig ein Zusammenhang mit akuten Erkrankungen, unerwünschten Arzneimittelwirkungen oder medizinischen Komplikationen. Die Diagnose wird klinisch gestellt und hängt vom Vorhandensein oder Fehlen bestimmter Merkmale ab. Angesichts der multifaktoriellen Ätiologie scheinen Multikomponenten-Ansätze am vielversprechendsten, um den Bedürfnissen der Betroffenen gerecht zu werden. Pharmakologische Interventionen haben sich weder zur Prävention noch zur Behandlung als klar wirksam erwiesen. Dieser Artikel gibt einen Überblick über das aktuelle klinische Wissens, einschließlich Ätiologie, Pathophysiologie, Diagnose, Prognose, Behandlung, Prävention und Verlauf.

## Introduction

The term delirium is derived from the Latin “de lira ire = to go off the rails” and was coined by Aulus Cornelius Celsus around 100 AD. As early as 500 years earlier, the *Corpus Hippocraticum* contained a description of two mental disorders that occur with high fever and severe physical illness: “phrenitis” (agitation) and “lethargus” (lethargy).

The term delirium is sometimes replaced in clinical practice by synonymous terms: organic brain syndrome or acute confusional state.

Delirium, defined as acute deterioration of cognitive function and attention, is a common mental disorder in geriatric patients, affecting up to 42% of those hospitalized [1]. Delirium determines dramatic consequences for geriatric patients: longer length of hospital stay, increased mortality, functional and cognitive deterioration, and increased need for institutional care [2].

## Symptomatology and epidemiology

Core symptoms include impairment of cognition and consciousness. Diagnostically groundbreaking are the inability to direct attention, limited perception of environmental stimuli, and inadequate reaction to the same. Amongst cognitive symptoms, perceptual and memory disorders are in the foreground, along with situational disorientation. Perceptual disturbances include misperceptions and visual, occasionally also scenic, hallucinations and paranoid symptoms. Psychomotor symptoms are often dominated by restlessness, but there may also be a pronounced hypoactivity, whereby a change between these manifestations is frequent. Based on the expression of psychomotor activity, hyperactive delirium contrasts with hypoactive delirium, in which the hypoactive variants are often misrecognized [3]. Up to 40% of affected patients show a mixed picture.

Delirium symptoms usually fluctuate over time and often aggravate in the early evening hours. In addition, there is often a considerably increased startle response, especially in connection with medical or nursing interventions.

By definition, the onset of delirium is acute to subacute (hours to days) and is often associated with the onset of physical illness. Duration is highly variable, ranging from a few hours to months, with a maximum total duration of 6 months by definition. Most often, delirious states resolve within 1–2 weeks.

According to Diagnostic and Statistical Manual of Mental Disorders (DSM 5), delirium is defined as follows [4]:

A. Disturbance of attention (i.e., reduced ability to direct, focus, sustain, and shift attention) and consciousness (reduced environmental orientation).
B. The disturbance develops within a short period of time (usually within hours to a few days), involves a change in the usual level of attention and consciousness, and tends to fluctuate in severity throughout the day.
C. In addition, there may be other cognitive symptoms (e.g., memory disturbance, disorientation, speech disturbance, disturbances in visuospatial abilities, or perception).
D. The disturbances in criteria A and C cannot be better explained by other preexisting or developing neurocognitive disorders (dementia); there is no context of a severe reduction in activity level, as in coma.
E. There is evidence from history, clinical examination, or laboratory findings that the disorder is a direct result of somatic disease, substance intoxication or withdrawal (e.g., addictive substances or medications), toxin exposure, or is a result of multiple etiologies.

## Pathogenesis and etiology

Delirium is a nonspecific acute brain failure with effects on psychopathology and behavior as a result of exogenous or endogenous factors. The widely accepted threshold concept of deliriogenesis postulates that the relationship between vulnerability and noxious agent plays the core role in development of delirium (Table 1). If vulnerability is high, a minor noxious agent is sufficient to trigger delirium and vice versa [5].

**Table 1:** Predisposing factors (A) and triggering agents (B) of delirium

*A: Predisposing factors defining vulnerability include*
Advanced age
Neurocognitive deficit (dementia), delirium in the medical history
Frailty (gerastenia)
Multimorbidity
Sensory disorders
Anemia
Malnutrition
Substance abuse
Depression
Social isolation
*B: Triggering (noxious) agents include*
Surgical interventions
Anticholinergic drugs
Psychoactive drugs (including antipsychotics, antidepressants, tranquilizers)
Intensive care unit
Re-surgery
Acute blood loss
Acute infections
Disturbances of electrolyte and water balance (i.e., hyponatremia, exsiccosis)
Sleep deprivation
Immobilization
Coercive measures, mechanical restraints
Withdrawal (drugs, alcohol)
Urinary catheter
Foreign environment

The relationship between delirium and advanced age has been demonstrated in numerous studies. Aging is characterized by the progressive loss of resources and adaptability, including brain function. However, it is unclear to what extent age per se is a risk factor or whether other factors associated with age, such as reduced health status, sensory impairment, multimorbidity, neurocognitive deficits, and polypharmacy, define the increased risk. Chronic renal, hepatic, cardiac, pulmonary, and central nervous system diseases play a significant role as risk factors in the context of multimorbidity [6]. Psychosocial stress can be of considerable importance; abrupt changes such as admission to a hospital or a nursing home can trigger delirium, as can lacking devotion, unprofessional caregiving, stimulus deprivation, stressful visitors, room changes, and the stress of examinations [7].

Numerous mechanisms have been hypothesized to contribute to the pathophysiology of delirium, including neurotransmitters, inflammation, electrolyte disorders, metabolic disturbances, physiologic stressors, and genetic factors [2].

In delirium in higher age, the search for a common terminal pathway often remains inconclusive due to complex multifactorial etiology. The systems’ integration failure hypothesis [8] integrates published concepts by describing the various results from each into an intricate network, thus emphasizing areas of similarities and intersections. The variable impact of these factors contributes to the development of the cognitive and behavioral symptoms of delirium.

At the neurotransmitter level, the *cholinergic system* appears to play a central role in the pathogenesis of delirium [9], and anticholinergic drugs therefore increase the risk of incident delirium. Serum levels of anticholinergic drugs have been shown to correlate with the extent of cognitive deficits, but an independent relationship of serum anticholinergic activity to the presence of delirium is questionable [10]. Anticholinergic delirium usually presents with motor hyperactivity, cognitive, and psychotic symptoms, and is associated with electroencephalogram (EEG) slowing. In addition, metabolic changes can affect cholinergic activity: hypoxic or hypoglycemic metabolic states increase the propensity to develop delirium, as does thiamine deficiency [9].

Anticholinergic agents include atropine, scopolamine, oxybutynin, tricyclic antidepressants, and benzodiazepines; opiates and nonsteroidal anti-inflammatory drugs (especially indomethacin) also bear an anticholinergic risk. Beta-lactam antibiotics, lithium, histamine H_2_ antagonists, diuretics, beta-blockers, antipsychotics, and quinolones have been reported to possess anticholinergic properties, as have theophylline and cardiac glycosides, with a pronounced dose-dependent effect for the latter.

The *dopaminergic system* also plays an essential role; agonists at D_1_ and D_2_ receptors increase the risk of delirium [11]. Dopaminergic substances such as L‑dopa, dopa agonists, and also bupropion and cocaine can therefore induce delirium. Dopaminergic mechanisms of action are also discussed for opiates and H_2_ antagonists.

In addition, there are interactions between cholinergic and dopaminergic transmission: anatomical and functional overlaps between these transmitters have also been shown within the cerebral cortex, so a subtle balance between these systems is prerequisite for intact cognitive performance. Moreover, the cholinergic system is also influenced by the activity of monoamines: dopamine, norepinephrine, and serotonin modulate both the sleep–wake cycle and the response to external stimuli [11].

Another relevant transmitter is *serotonin*. For different serotonin receptors and different brain regions, cholinergic deficits could be associated with both serotonergic deficits and serotonergic excess. In addition, serotonin can also inhibit cholinergic transmission via dopaminergic activation. Clinically significant is the *serotonin syndrome*, which occurs in association with the administration of selective serotonin reuptake inhibitors (SSRIs). Symptoms include tremor, hyperreflexia, spontaneous clonus, muscle rigidity, ocular clonus, agitation, and fever. Tricyclic antidepressants, opiates, antibiotics, fluconazole, antiemetics, triptans, dextromethorphan, and monoamine oxidase (MAO) inhibitors serve as aggravating co-medications [12].

Further neurotransmitters potentially involved in the pathogenesis of delirium include *glutamate* and *gamma-amino-butyric acid (GABA).* A decrease in GABAergic stimulation is likely to be the central mechanism of delirium after benzodiazepine withdrawal.

It should be emphasized that the individual transmitters unfold multiple interactions at different cortical and subcortical levels, with cholinergic deficit and dopaminergic excess considered as major common end routes.

The pathogenetic role of endogenous hormones and neuromodulators is of increasing interest [13, 14], offering new therapeutic options.

The release of norepinephrine via the sympathetic nervous system is common in the stress response, causing increased release of glucocorticoids via the hypothalamic–pituitary–adrenocortical axis, thus also contributing to glial cell activation and neuronal damage [15].

In addition to neurotransmitters, inflammatory processes play a central role in the development of delirium. Disorders occurring outside the brain, such as inflammation, trauma, or surgery, can therefore also trigger delirium. In the context of a systemic inflammatory reaction, cytokines are released, which cross the blood–brain barrier and, by activating microglial cells releasing proinflammatory cytokines, cause an inflammatory reaction in the brain with damage to neurons. In addition to this direct neurotoxic effect, cytokines can also cause disruption of neurotransmitter synthesis and release [16].

Due to frequent polypharmacy in the elderly, medications play a major role as triggers: 12–39% of all delirium cases in the elderly may be classified as pharmacogenic [17]. In general, polypharmacy, i.e., taking five or more medications, should be considered a relevant risk factor for delirium. Age-related changes important for adverse drug effects include the reduced elimination capacity of kidneys and liver, the decrease of water, lean body mass and albumin, and the increase of body fat percentage.

A number of substances with central nervous effects (antidepressants, antipsychotics, antiepileptics) are known to lead to retention of free water via an antidiuretic hormone (ADH) effect and thus to *hyponatremia*, which is often a cofactor of delirious syndromes; antidiabetic drugs may contribute by inducing hypoglycemia.

## Diagnosis

The diagnosis of delirium is primarily by clinical means: detailed exploration and observation as well as physical examination are indispensable.

Diagnostic clues are:

- Inability to focus attention.
- Loss of the ability to think with the usual clarity and coherence.
- Limited perception of environmental stimuli and inadequate response to them.
- Cognitive disturbances such as perceptual and memory disturbances, often striking situational disorientation.

Attention can be tested simply by asking the patient to enumerate the months backward beginning with December (reaching July should at least be possible correctly), or to spell the word “radio” backward.

In the context of acute hospital admission, a standardized delirium screening, e.g., with the validated *Delirium Observatie Screening Schaal* (DOS scale), should be performed for all patients who are over 70 years of age [18]. Screening with a validated instrument allows the detection of incident delirium with high sensitivity and specificity and should be performed by the nursing staff once per shift to detect fluctuations and acute changes [19].

Those identified as positive in the screening should be rapidly referred to a definitive diagnosis. The criteria according to DSM 5 or International Statistical Classification of Diseases and Related Health Problems (ICD-10) are suitable for this purpose; the Confusion Assessment Method (CAM), which is also recommended for emergency situations, is widely used as an assessment tool. It includes the relevant features 1) acute onset, 2) fluctuating course, 3) disturbance of attention, 4) distracted thinking, and 5) disturbance of consciousness. A diagnosis should be made if features 1–3 and additionally either 4 or 5 are present. Sensitivity and specificity are both very high at 95% [20].

To detect patients at risk, besides medical history, multivariable prediction models may be engaged to calculate risk estimates from data from previous hospital stays and the current admission [21].

If the cause of delirium is unclear, a somatic cause must be clarified as soon as possible. This is also necessary if, for example, delirium occurs after a clear interval in the first days after a surgical intervention. (Third party) medical, medication, and drug history are of importance; special attention should be paid to central nervous system (CNS)-active substances and alcohol. Physical examination includes somatic and neuropsychological status; lab values include blood glucose, electrolytes, liver and kidney function, blood count, cardiac enzymes, urinary status, thyroid hormones, and inflammatory parameters. Examination of abdomen (urinary retention, ileus) and of bones and joints is mandatory, since pain due to fractures may be causative. Radiographs and ultrasound may complement the physical exam, electroencephalography (EEG) serves to rule out a nonconvulsive status epilepticus, and cerebrospinal fluid (CSF) is analyzed if an infection of the central nervous system is suspected. Examinations that do not promise any therapeutic consequences should be avoided, as they may cause additional stress for the patients.

## Prevention

Because of its deleterious consequences, prevention of delirium is of paramount importance. Consistent nonpharmacological multicomponent management according to a protocol that controls risk factors such as sleep deprivation, immobility, sensory deficits, pharmacotherapy, and dehydration has been shown to reduce the delirium risk by up to 30%, and early transfer to outpatient rehabilitation can also significantly reduce delirium incidence [22]. Treatment in a specialized geriatric unit reduces the absolute risk by 20% and shortens the average duration of delirium by 5 days. In the surgical setting, proactive geriatric consultation was shown to reduce the incidence of delirium from 50% to 28% after hip fractures in a randomized controlled trial. Recommendations included adequate oxygenation, correction of fluid and electrolyte imbalances, treatment of pain, discontinuation of unnecessary medications, early removal of bladder catheters, adequate caloric intake, early mobilization and rehabilitation, early recognition and treatment of postoperative complications, avoidance of sensory overstimulation, and pharmacological treatment for hyperactive delirium [23]. Isolated prodromal syndromes occur in hip fractures up to 4 days before full-blown delirium and allow adequate intervention if identified in a timely manner [24]. Dementia patients who are particularly at risk of delirium should be offered constant accompaniment by their family or other close caregivers. This requirement means that older, multimorbid, cognitively impaired people should be provided with a contact person (“sitter”) from admission to discharge, who accompanies them through all examinations and transfers ([25]; Table 2).

**Table 2:** Recommendations for the prevention of delirium

Prevention of delirium
Avoid causal factors: unnecessary hospitalization, polypharmacy
Timely recognition of prodromal symptoms: agitation, vivid dreams, insomnia, hallucinations
If inpatient admission is necessary, the patient should receive qualified geriatric care right from the start, i.e., in perioperative management
Dementia patients should be offered constant accompaniment by their family or other close caregivers (“sitters”)
Consistent delirium screening, assessment of dementia, depression, anxiety disorders, addictive disorders (alcohol, benzodiazepines, nicotine), identification of history of delirium, geriatric consultation, and medication review are recommended
Minimizing stress, giving time for questions, and optimal pain management are also recommended for the perioperative setting

## Medication for prevention?

Against the background of the abovementioned multitude of risk factors (predisposition of the patient, variety of delirium-inducing noxae), the complexity of delirium development becomes clear. A valid recommendation for pharmacological prevention cannot be given at present, even before elective surgical interventions. This underlines the high importance of nonpharmacological intervention.

Pharmacologically, the nightly administration of melatonin showed preventive effects in older—predominantly internal medicine—patients, whereas a high-quality study in 459 patients showed no effect on the incidence of delirium after near-hip fracture [26]. A recent meta-analysis concludes that perioperative melatonin and melatonergic agents may have no effect on the prevention of postoperative delirium [27]. Cholinergics such as donepezil (more side effects than placebo) and rivastigmine (no effect in cardiac surgery patient group) were also disappointing; data on antipsychotics (haloperidol, olanzapine, risperidone) are also inconsistent. Low-dose haloperidol prophylaxis should be considered at the most in individual cases in patients at a high risk of delirium [28], but general pharmacologic prevention is not recommended [29].

## Treatment

The basis of treatment is observation, reassurance, and attendance; a sitting or walking guard is in any case preferable to restraint.

Identification and treatment of underlying diseases and discontinuation of high-risk medications is imperative. Appropriate laboratory diagnosis for fluid and electrolyte balancing, antibiotic therapy in cases of suspected infection, therapeutic nursing orientation support, adequate lighting, explanation of diagnostic and therapeutic steps, as well as avoidance of transfers, unnecessary noise, and visual overstimulation are the measures of choice [30]. Distracting is better than confronting; continence management, prevention of pressure ulcers and falls, and early mobilization are of proven benefit [2, 31]. Restraints should be avoided, as they can promote agitation, oversedation is also fraught with complications (falls, pneumonia).

## Pharmacological therapy

Pharmacological treatment is necessary in cases of hyperactive delirium, anxiety, and agitation. The pharmacological treatment should be based on the cluster of symptoms presented and comorbidities.

### Antipsychotics (neuroleptics):

Interestingly, despite the significant incidence of delirium in hospitalized patients, there is no uniformly accepted drug intervention. No significant difference in efficacy and safety was shown between typical and atypical antipsychotics. The authors of a comprehensive analysis concluded that current evidence does not support the superiority of atypical antipsychotics over haloperidol [32, 33]. Low-dose haloperidol (0.5–3.0 mg per day, maximum 3–5 days) as well as atypical antipsychotics resulted in a reduction in delirium scores without significant differences between the agents. Low-dose haloperidol did not show a higher incidence of side effects, while dosages of > 4.5 mg/d caused more frequent extrapyramidal side effects compared to atypical antipsychotics. Risperidone (0.5–3 mg/d) is also widely used—especially for delirium in the context of Alzheimer’s. Quetiapine (25–300 mg/d) is recommended for delirium and hallucinosis in the context of Parkinson’s disease due to the low incidence of extrapyramidal side effects.

When using antipsychotics, potential side effects on the cardiovascular system (QTc time), glucose metabolism, risk of falls, and extrapyramidal motor function must be taken into account. In addition, increased mortality rates have been reported with antipsychotics, especially in dementia patients [34]. Consistent weighing of the potential risk/benefit ratio and monitoring (i.e., ECG) are obligatory. For intravenously administered haloperidol (off-label!), the U.S. Food and Drug Administration (FDA) has issued a “warning” because of the risk of QTc prolongation and development of torsades de pointes.

### Benzodiazepines:

Short-acting benzodiazepines such as lorazepam 3 × 0.5 to 3 × 1 mg are widely used in the treatment of delirium, but the evidence is based on only a few adequate-quality studies, mainly in delirium associated with substance abuse and withdrawal. The authors of a Cochrane review note that increased and protracted sedation may actually worsen the condition of delirious patients when treated with lorazepam [35]. An increased risk of falls is also associated with benzodiazepine administration. Furthermore, benzodiazepines can transform hyperactive delirium into hypoactive delirium. Benzodiazepines (BZD) are chosen in hyperactive delirium associated with alcohol or drug withdrawal, severe cardiac failure, or Parkinson’s disease.

### Trazodone:

A retrospective medical chart review showed similar results for trazodone and quetiapine in terms of improvement of delirium symptoms [36]. In a prospective study in palliative cancer patients, low-dose trazodone proved generally safe and reduced delirium severity [37].

The occurrence of delirium in the palliative situation is common, accompanying anxiety can be treated with BZD or pregabalin. The antiepileptic *pregabalin* ameliorates neuropathic pain and anxiety and was shown to reduce postoperative opioid consumption and the incidence of confusion after heart surgery in elderly patients [38]. However, high-quality evidence to confirm these results is lacking.

As soon as the causal therapeutic measures have taken effect, antipsychotic or sedative treatment should be discontinued. Target symptoms of psychopharmacotherapy, lack of efficiency of initially implemented nonpharmacological measures, clinical course, and dose reduction attempts must be recorded.

## Delirium in the intensive care unit

The care of delirious patients is common in intensive care units (ICUs), occurring in up to 80% of patients [39]. Delirium is either triggered by the acute illness itself or by the intensive care environmental conditions. A meta-analysis of data from more than 16,000 patients underscores the high relevance of intensive care delirium: the risk of mortality during hospitalization and afterwards is more than doubled, the length of stay in the intensive care unit and in the normal ward is prolonged, as is the duration of ventilation. Cognitive impairment is found more frequently in affected patients both 3 and 6 months after hospitalization [40]. Validated scales are available for delirium screening and monitoring in the ICU, the version of the Confusion Assessment Method for the Intensive Care Unit (CAM-ICU) being most broadly established, which can be performed in intubated patients [41]. Without standardized screening, more than 70% of affected delirious patients are not recognized as such; at the same time, monitoring with a validated instrument is associated with improved outcome in geriatric patients. In summary, regular evaluation of sedation depth, analgesics, and delirium results in fewer nosocomial infections, shortened duration of ventilation and intensive care, and reduced mortality [42]. A recent systematic review and network meta-analysis showed superiority for dexmedetomidine compared to placebo and antipsychotics with respect to the occurrence of delirium and the length of ICU stay [43].

## Prognosis

Delirium may recover completely, but also with a defective state, depending on the underlying disease. The mortality of 25–33% in the acute phase is similar to that of acute myocardial infarction or sepsis [44]; 25% of all older hospitalized delirious patients die within 3 to 4 months of diagnosis, although only part of this excess mortality can be explained by the underlying diseases [45]. Delirium causes an increased risk of falls and infection and often leads to a permanent deterioration in everyday competence and neurocognitive performance: 38 months after delirium, 53.8% of those affected showed cognitive deficits [44]. The more severe and prolonged the delirium, the more frequent and severe the sequalae—it is therefore essential to detect and treat delirium early.

Delirium due to metabolic and toxic causes is prognostically more favorable than delirium in dementia: many of these patients are hospitalized longer, suffer more complications, and are more likely to be admitted to a nursing home.

In addition, the relationship with frailty should be noted. Delirium is a risk factor for frailty (gerastenia), and those who are frail are at high risk of experiencing delirium [2]. Frailty and delirium share many similarities, so prevention of delirium can also be seen as prevention of progression to frailty. Both lead to deterioration in general condition, daily living skills, and cognitive function. Both entities have identical predisposing factors such as malnutrition, sarcopenia, systemic inflammation, neuroendocrine dysregulation, oxidative stress, or mobility limitations, and are prototypical of multidimensional geriatric syndromes [46]. The increased risk of experiencing delirium in older age and frailty was also demonstrated during the COVID-19 pandemic, as was the dramatic impact of delirium on mortality [47].

## Conclusion

Delirium is a common and serious condition in geriatric patients. Early detection is crucial for adequate therapy, with nonpharmacologic management and treatment of triggering conditions as cornerstones, whereas pharmacologic treatment remains controversial.

Prevention of this potentially life-threatening problem includes recognition of patients at risk, avoidance of causal factors, and timely response to prodromal symptoms. Current knowledge does not support pharmacological measures for prevention.
